# Supplementary material for: Strain-level antigen variation facilitates immune evasion in Bacteroides thetaiotaomicron
Source: J Immunol. 2026 Mar 18;215(3):vkaf333. doi: 10.1093/jimmun/vkaf333 (PMC13017159; doi:10.1093/jimmun/vkaf333)
Supplement: vkaf333_Supplementary_Data [file vkaf333_supplementary_data.zip › JIMMUNOL-RA-2025-004_supplemental_figures.pdf]

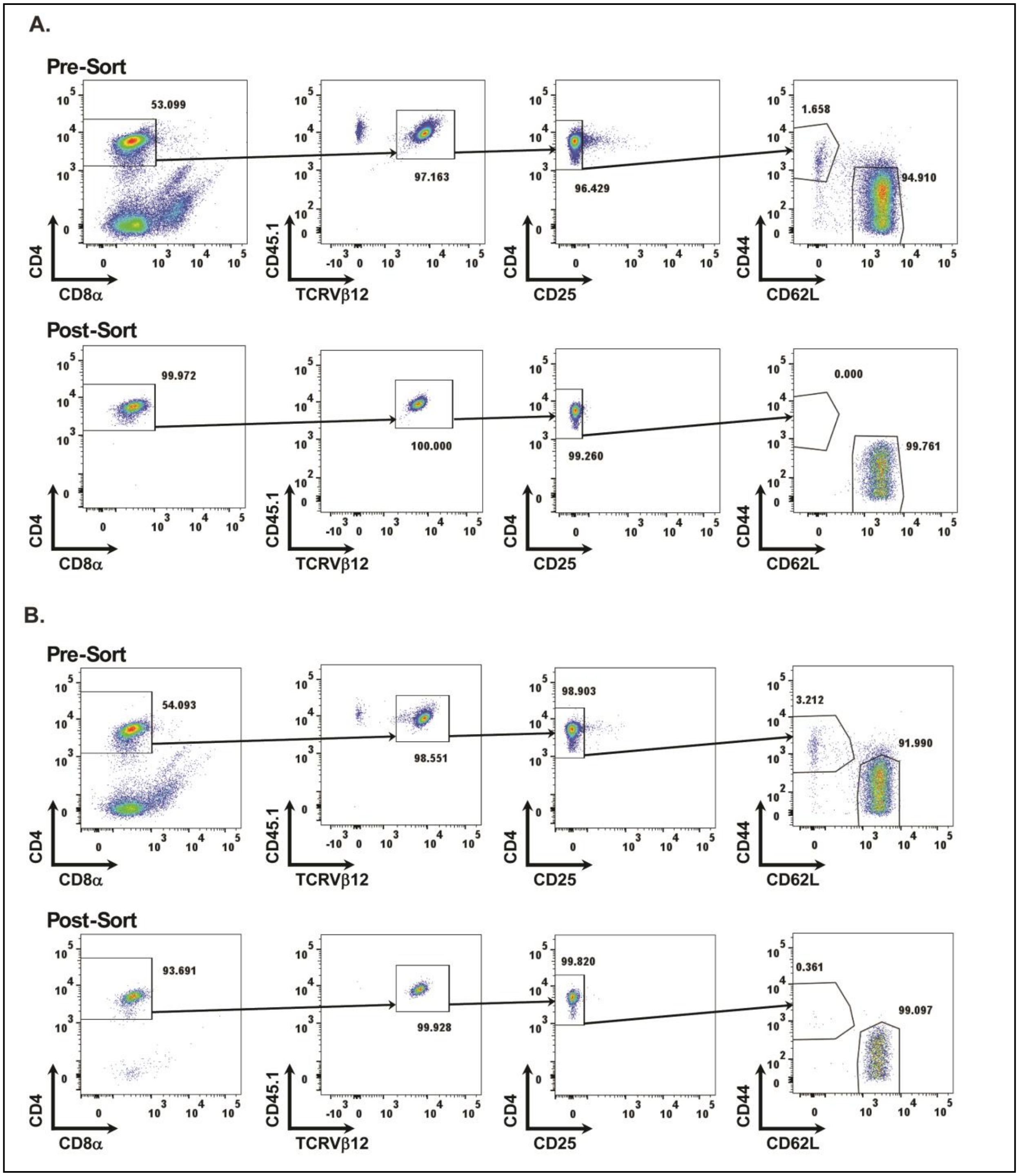

Supplementary Figure 1-Gating strategies for purification of cells by flow cytometric based cell sorting

Naïve BθOM CD4+ T cells for *in vivo* adoptive transfer experiments were purified by flow cytometric based cell sorting. Representative pre- and post- sort frequencies are shown from two independent experiments.

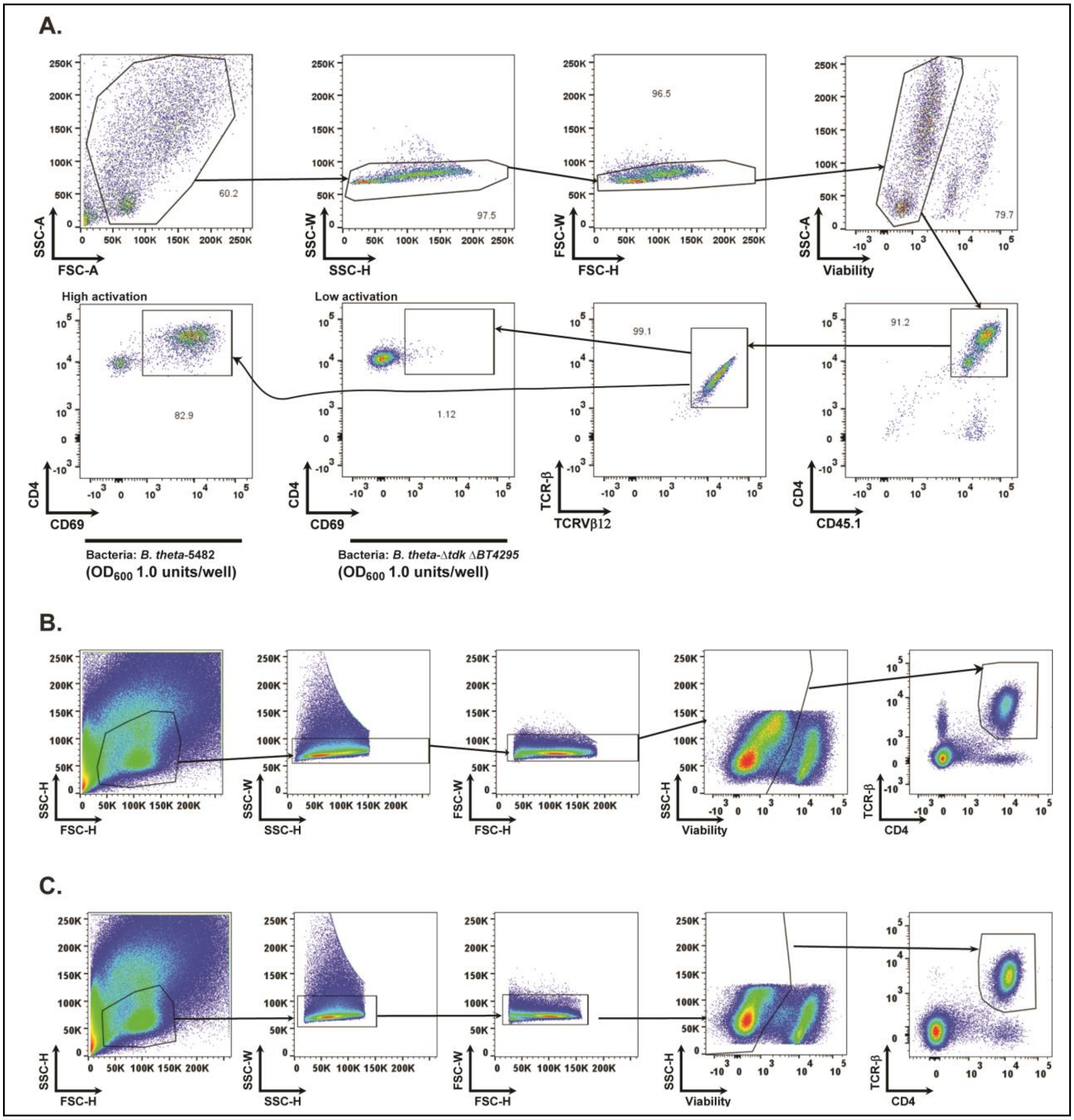

Supplementary Figure 2-Gating strategies for purification and analysis of BθOM CD4+ T cells by flow cytometric based cell sorting and flow cytometry respectively.

BθOM CD4+ T cell activation following *in vitro* stimulation or following *in vivo* adoptive transfer was assessed via flow cytometry. Shown are representative gating strategies used to phenotype BθOM CD4+ T cells from these assays.

(A) Strategy for assessment of *in vitro* stimulation of BθOM CD4+ T cells
(B) Strategy for assessment of BθOM CD4+ T cells in experiments comparing *B. theta*-VPI-5482 and *B. theta*-dnLKV9 *in vivo*.
(C) Strategy for assessment of BθOM CD4+ T cells in experiments comparing *B. theta*$^{\Delta tdk \Delta BT4295::BT4295}$ (WT) *or B. theta*$^{\Delta tdk \Delta BT4295::T548s}$ (T548S) *in vivo*.

2
